# Ultrasensitive and visual detection of Feline herpesvirus type-1 and Feline calicivirus using one-tube dRPA-Cas12a/Cas13a assay

**DOI:** 10.1186/s12917-024-03953-9

**Published:** 2024-03-16

**Authors:** Fumei Jiang, Yunjia Liu, Xiaonong Yang, Yan Li, Jian Huang

**Affiliations:** 1 Key Laboratory of Animal Medicine, Southwest Minzu University, Chengdu City, Sichuan Province China; 2 Veterinary Teaching Hospital, Southwest Minzu University, Chengdu, China; 3 Department of Clinical Veterinary Medicine, College of Animal Science and Veterinary Medicine, Southwest Minzu University, No. 16, South 4th Section, 1st-Ring Road, Wuhou, Chengdu, Sichuan 610041 China

**Keywords:** FHV-1, FCV, Simultaneous detection, RPA, Hybrid CRISPR/Cas effectors, Visual modality

## Abstract

**Background:**

Feline herpesvirus type 1 (FHV) and Feline calicivirus (FCV) are the primary co-infecting pathogens that cause upper respiratory tract disease in cats. However, there are currently no visual detection assays available for on-site testing. Here, we develop an ultrasensitive and visual detection method based on dual recombinase polymerase amplification (dRPA) reaction and the hybrid Cas12a/Cas13a *trans*-cleavage activities in a one-tube reaction system, referred to as one-tube dRPA-Cas12a/Cas13a assay.

**Results:**

The recombinant plasmid DNAs, crRNAs, and RPA oligonucleotides targeting the FCV ORF1 gene and FHV-1 TK gene were meticulously prepared. Subsequently, dual RPA reactions were performed followed by screening of essential reaction components for hybrid CRISPR-Cas12a (targeting the FHV-1 TK gene) and CRISPR-Cas13a (targeting the FCV ORF1 gene) *trans*-cleavage reaction. As a result, we successfully established an ultra-sensitive and visually detectable method for simultaneous detection of FCV and FHV-1 nucleic acids using dRPA and CRISPR/Cas-powered technology in one-tube reaction system. Visual readouts were displayed using either a fluorescence detector (Fluor-based assay) or lateral flow dipsticks (LDF-based assay). As expected, this optimized assay exhibited high specificity towards only FHV-1 and FCV without cross-reactivity with other feline pathogens while achieving accurate detection for both targets with limit of detection at 2.4 × 10^− 1^ copies/μL for the FHV-1 TK gene and 5.5 copies/μL for the FCV ORF1 gene, respectively. Furthermore, field detection was conducted using the dRPA-Cas12a/Cas13a assay and the reference real-time PCR methods for 56 clinical samples collected from cats with URTD. Comparatively, the results of Fluor-based assay were in exceptional concordance with the reference real-time PCR methods, resulting in high sensitivity (100% for both FHV-1 and FCV), specificity (100% for both FHV-1 and FCV), as well as consistency (Kappa values were 1.00 for FHV-1 and FCV). However, several discordant results for FHV-1 detection were observed by LDF-based assay, which suggests its prudent use and interpretaion for clinical detection. In spite of this, incorporating dRPA-Cas12a/Cas13a assay and visual readouts will facilitate rapid and accurate detection of FHV-1 and FCV in resource-limited settings.

**Conclusions:**

The one-tube dRPA-Cas12a/Cas13a assay enables simultaneously ultrasensitive and visual detection of FHV-1 and FCV with user-friendly modality, providing unparalleled convenience for FHV-1 and FCV co-infection surveillance and decision-making of URTD management.

**Supplementary Information:**

The online version contains supplementary material available at 10.1186/s12917-024-03953-9.

## Background

Upper respiratory tract disease (URTD) is a prevalent condition that affects cats of all ages [1]. Feline patients exhibit symptoms like sneezing, conjunctivitis, and nasal or ocular discharge, ranging from mild to severe illnesses, which is commonly caused by viral or bacterial agents, such as feline herpesvirus type-1 (FHV-1), feline calicivirus (FCV), *Mycoplasma felis*, *Chlamydia felis*, and *Bordetella bronchiseptica* [1, 2]. Additionally, several studies also sporadically documented the relationships between URTD and feline panleukopenia virus (FPV), feline coronavirus (FCoV), *Escherichia coli* or *Klebsiella pneumoniae*, etc. [2–5]. Increasing epidemiological investigations have revealed that co-infection of FHV-1 (the order of *α-Herpesvirinae* and the family of *Herpesviridae*, DNA virus) and FCV (the order of *Picornavirales* and the family of *Caliciviridae*, RNA virus) accounts for over 20 ∼ 50% of URTD cases in multi-cat household or high-stress environments [6, 7], potentially leading to reactivation of FCV or FHV-1 in feline carriers and reinfection [8, 9]. Moreover, the reduced virus shedding in mildly infected cats or asymptomatic individuals challenge to the sensitivity and efficiency of current molecular diagnostics targeting FHV-1 and FCV in veterinary practices. Therefore, there is an urgent need for the development of an ultrasensitive diagnostic approach suitable for on-site detection and early surveillance of FHV-1 and FCV infection.

Various real-time PCR methods for detecting FHV-1 or FCV have been well developed [6, 10–13], but their clinical applications is limited due to the requirement for well-trained personnel, PCR amplification using a thermal cycler, and time-consuming processes especially for real-time detection. Therefore, novel methodologies for rapid nucleic acid detection based on isothermal amplification technology, such as recombinase polymerase amplification (RPA) or its variant [14, 15], have emerged as robust alternatives for the rapid detection of FHV-1 or FCV nucleic acids. However, the potential non-specific amplification may compromise the specificity of these detection methods. To address this concern, CRISPR-based (Clustered regularly interspaced palindromic repeats) biosensors coupled with RPA have been employed to detect epidemic FHV-1 or FCV variants [16, 17], which have demonstrated higher sensitivity compared to the real-time PCR technology [16, 18] and other isothermal amplification approaches [14, 15]. In the integrated RPA/CRISPR-Cas reaction system, the designed guide RNA (gRNA) will specifically recognize target gene sequence (RPA products), thereby activating the *trans*-cleavage activity of CRISPR-Cas nucleases to generate detectable signals by cleaving ssDNA or ssRNA reporters [16, 17]. Nevertheless, simultaneous detection of FHV-1 and FCV nucleic acid has not yet been achieved in a one-tube CRISPR-powered reaction system.

CRISPR-Cas12a and CRISPR-Cas13a orthologs, such as LbaCas12a and LwaCas13a, derived from diverse bacterial species, are commonly utilized for molecular detection. Accordingly, the Cas12a-based DETECTR (DNA Endonuclease-Targeted CRISPR Trans Reporter) [19] and Cas13a-based SHERLOCK (Specific High Sensitivity Enzymatic Reporter Unlocking) platforms [20] have been developed and extensively employed as diagnostic tools. In a previous study, the cost-effective and efficient approach was developed by exploiting Cas13 homologs (Cas13a and Cas13b) and Cas12a to enable multiplexed viruses discrimination in a single system [21]. However, complete elimination of fluorescence interference in multiple Cas13-powered system remains an ongoing challenge, which may possibly result in ambiguous interpretation when using fluorescence or colorimetric visual readouts. In another study, a lateral flow assay incorporating CRISPR/Cas9 with multiplex RT-RPA was established to simultaneously detect the E and Orf1ab genes of SARS-CoV-2 while reduced detection capability for suspected clinical samples (cycle threshold values over 36) was observed [22]. Recently, LbCas12a and LbuCas13a were assembled to detect the N and Orf1ab genes of SARS-CoV-2 using a handheld fluorescence detector [23], which generated specific fluorescence readouts without signal interference. Nevertheless, the detection efficiency for dual genes in several clinical samples was not consistent compared to the RT-qPCR methods [23], suggesting the need for further improvement to achieve balanced and saturated fluorescence signals for multiple targets detection. Additionally, a hybrid CRISPR-Cas12a/Cas13a system has also been successfully developed for duplex detection of SARS-CoV variants (N and Orf1ab gene), which was combined with multiplex lateral flow assay. [24]. This innovative approach provided adevice-independent alternative for multiple genes detection. Moreover, similar hybrid CRISPR-Cas system has been successfully applied to detect crop pathogens (cauliflower mosaic virus and *rhizobium radiobacter*), human papillomaviruses (HPVs), or exosomal proteins coupling with multiplex-PCR or multiplex-RPA assays [25–28]. Although these findings highlight the flexibility of hybrid CRISPR-Cas system adapted for various targets detection. Further efforts are needed to enhance the efficiency for detecting the targets of interest regarding the kinetic equilibrium of molecular determinants involved in the hybrid reaction system.

 In this study, we developed a novel approach, referred to as the dRPA-Cas12a/Cas13a assay, by integrating dual RPA (dRPA) reactions with a hybrid Cas12a/Cas13a trans-cleavage system for simultaneous detection of FHV-1 and FCV nucleic acids, and validated its detection efficacy in clinical settings.

## Results

### Dual RPA reaction

To assess the efficacy of the RPA reaction, purified products obtained from single RPA and dual RPA were submitted for 2% gel electrophoresis analysis. The dual RPA reaction yielded two distinct products as single RPA reaction did with high efficiency (Supplementary Fig. 1), which provided both FHV-1 TK gene and FCV ORF1 gene templates for subsequent utilization in one-tube CRISPR-Cas12a/Cas13a *trans*-cleavage system.

### Visual Readouts of one-tube dRPA-Cas12a/Cas13a assay

**Fig. 1:**
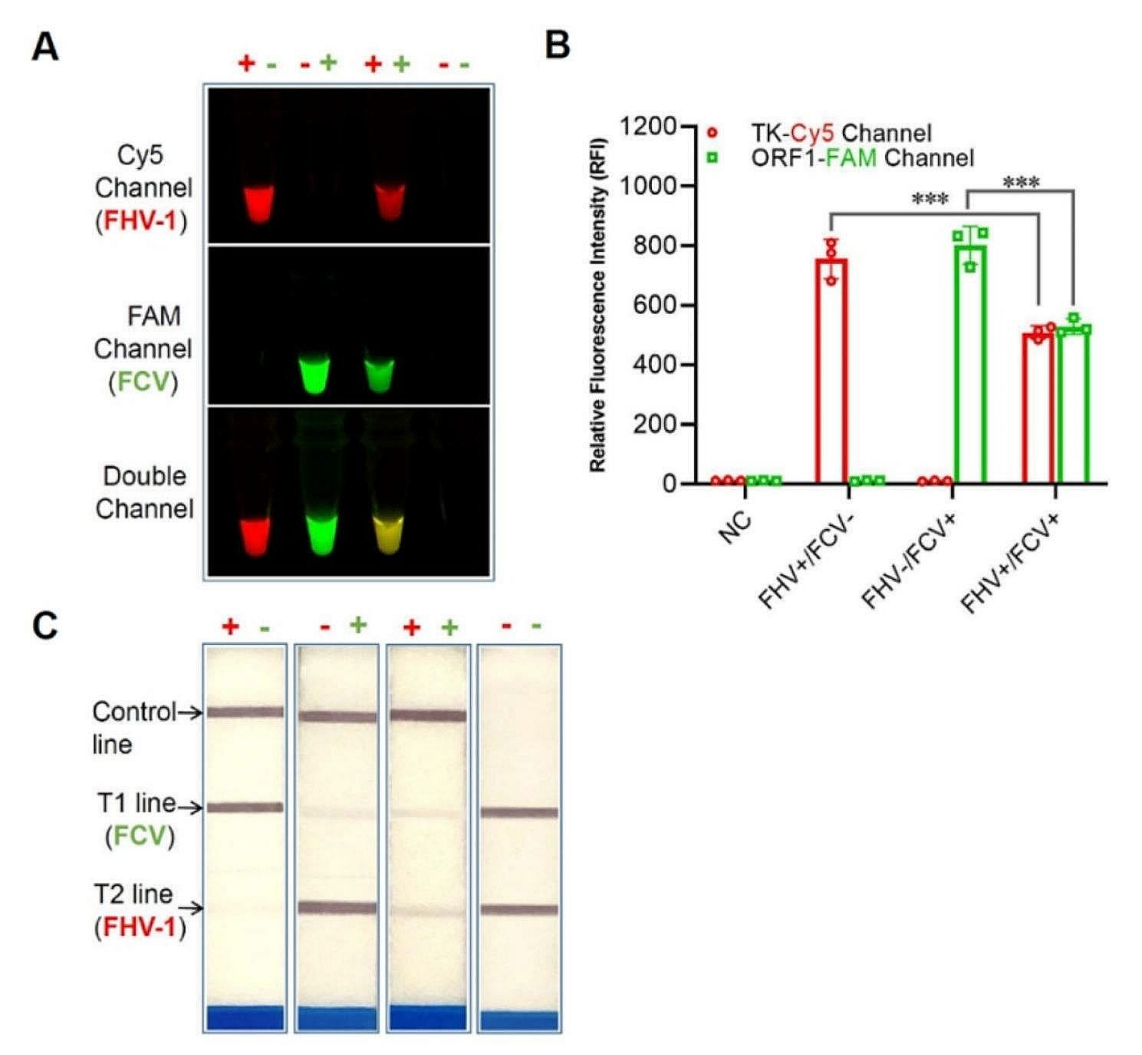
Preliminary establishment of dRPA-Cas12a/Cas13a assay. Positive detection readouts for single FHV-1 gene, single FCV gene, and double targets were obtained using preliminary dRPA-Cas12a/Cas13a assay without signal disturbance by fluorescence (**A**) and lateral flow dipstick modality (**C**), while unoptimized duplex reaction system resulted in reduced fluorescence intensity for both targets compared to initial single target (**B**). NC: negative control (DNase/RNase-free water). The statistical significance indicated by asterisks, ***P < 0.001

To assess the performance of the one-tube dRPA-Cas12a/Cas13a assay, the detection results were visualized using a multi-color fluorescence detector (Fluor-based assay) and lateral flow dipstick (LDF-based assay), respectively. As the Cas12a and Cas13a *trans*-cleavage activities were efficiently triggered by specific crRNA-target recognition, detectable signals in different exciting light channels or lateral flow dipsticks were generated. It was expected that detection signals of FCV ORF1 gene and FHV-1 TK gene were observed in single channel or double channel within the one-tube reaction systems, demonstrating no mutual interference between Cas12a and Cas13a-based reactions (Fig. 1). Notably, reduced fluorescence intensity occurred in both fluorescence channels in the preliminary one-tube reaction system.

### Optimization of one-tube dRPA-Cas12a/Cas13a assay

The one-tube dRPA-Cas12a/Cas13a assay successfully achieved simultaneous detection of dual targets in the preliminary reaction system, but reduced detection capabilities for both targets were still observed. Therefore, we optimized several crucial components of one-tube reaction systems to obtain a maximum detection capability equivalent to the corresponding single assay. As shown in the results, increasing amounts of dual RPA products enhanced fluorescence intensity and resulted in balanced fluorescence signals in serial screenings. Among them, 4 μL of dual RPA products were determined as the optimal and cost-efficient amount in the 30 μL one-tube reaction systems (Supplementary Fig. 2). To investigate whether competitive inhibition between Cas12a and Cas13a activities would occur and subsequently impair the detection capabilities for both targets, we tailored the amounts of RNase inhibitor and T7 RNA polymerase mix (the exclusive components for Cas13a-based assay). As expected, increasing amounts of RNase inhibitor enhanced Cas13a activity while slightly inhibiting Cas12a activity, while additional amounts of RNase inhibitor (over 24 U) didn’t have remarkable effect on both fluorescence signals (Supplementary Fig. 3). Furthermore, gradual increments of T7 RNA polymerase mix (25 ∼ 100 U) could promote the Cas13a activity. However, its further accumulation (125 ∼ 150 U) in the one-tube reaction systems significantly compromised the Cas12a activity (Supplementary Fig. 4). Finally, the optimal amounts for RNase inhibitor and T7 RNA polymerase mix were determined as 24U and 100 U, respectively, in current one-tube dRPA-Cas12a/Cas13a assay.

### Specificity and sensitivity of one-tube dRPA-Cas12a/Cas13a assay

**Fig. 2:**
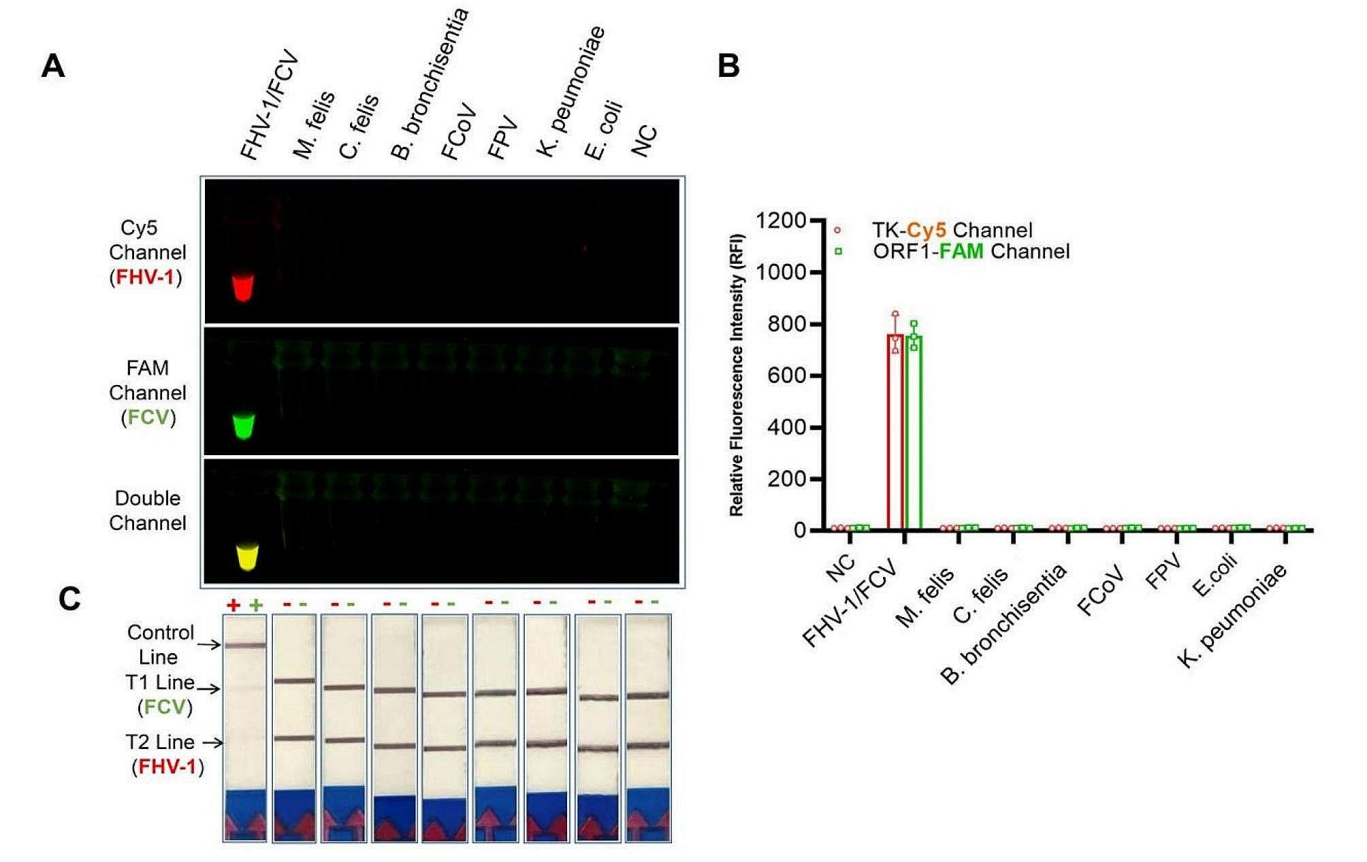
Specificity of dRPA-Cas12a/Cas13a assay. (A) The FHV-1, FCV, and feline-associated pathogens were detected using optimized dRPA-Cas12a/Cas13a assay. This assay only detected FHV-1 and FCV simultaneously by fluorescence (**A**) and lateral flow dipstick (**C**) modality, and no cross-reaction with other feline pathogens was found. (**B**) The balanced fluorescence signals for FHV-1 and FCV was observed in the one-tube reaction systems. NC: negative control (DNase/RNase-free water)

**Fig. 3:**
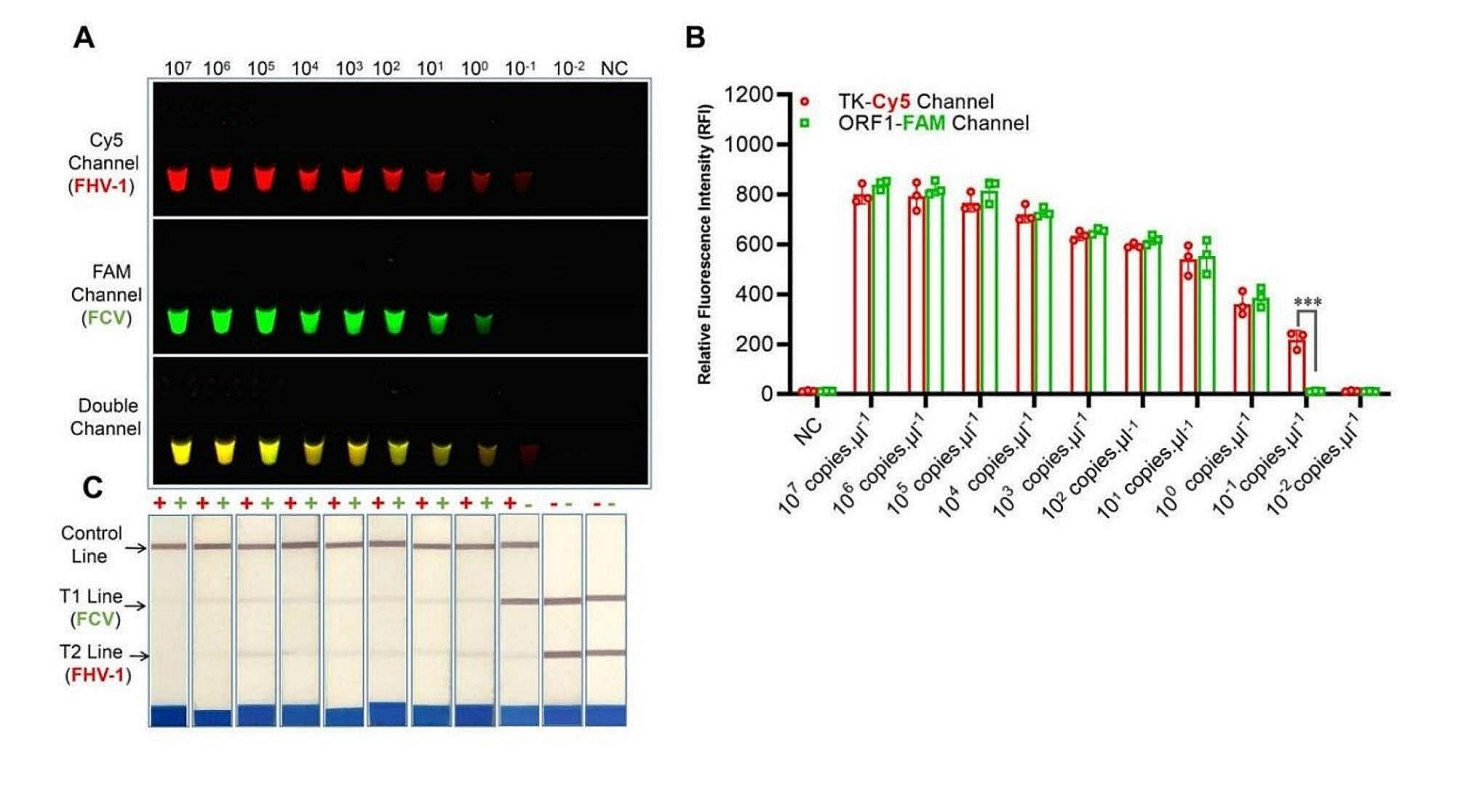
Sensitivity of dRPA-Cas12a/Cas13a assay. Serial dilutions (10^7^-10^− 2^) of FHV-1 and FCV plasmid DNA were tested using optimized dRPA-Cas12a/Cas13a assay. The limit of detection (LOD) for FHV-1 and FCV were at 10^− 1^ and 10^0^ dilution, respectively, by fluorescence (**A**) and lateral flow dipstick (**C**) modality. (**B**) The detection capabilities for FHV-1 and FCV were nearly parallel in serial dilutions, while the LOD for FHV-1 (10^-1^ dilution) was lower than FCV (10^0^ dilution). The statistical significance was indicated by asterisks, ***P < 0.001. NC: negative control (DNase/RNase-free water)

The specificity and sensitivity of the optimized one-tube assay were further tested, respectively. As a result, the optimized one-tube assay exclusively detected nucleic acids of FHV-1 and FCV, exhibiting no cross-reactivity with other feline pathogens. (*M. felis*, *C. felis, B. bronchiseptica*, FCoV, FPV, *K. peumoniae* and *E. coli*) (Fig. 2). The initial concentration of FHV-1 and FCV plasmid DNA were previously determined as 2.35 × 10^7^ copies/μL and 5.5 × 10^7^ copies/μL, respectively. In serial dilution testings, the limit of detection (LOD) for FHV-1 and FCV were 2.4 × 10^− 1^ copies/μL and 5.5 copies/μL, respectively (Fig. 3).

### Clinical detection and concordance with reference real-time PCR methods

**Table 1:** Concordance analysis between dRAP-Cas12a/Cas13a-Fluor assay and real-time PCR methods

		FHV-1 qPCR		Total	Sensitivity (%)	Specificity (%)	Kappa
		+	-				
dRPA-Cas12a/13a-Fluor assay	+	19	0	19	100	100	1.00
	-	0	37	37			
Total		19	38	56			
		FCV RT-qPCR					
		+	-				
dRPA-Cas12a/13a-Fluor assay	+	21	0	21	100	100	1.00
	-	0	35	35			
Total		21	35	56			

**Fig. 4:**
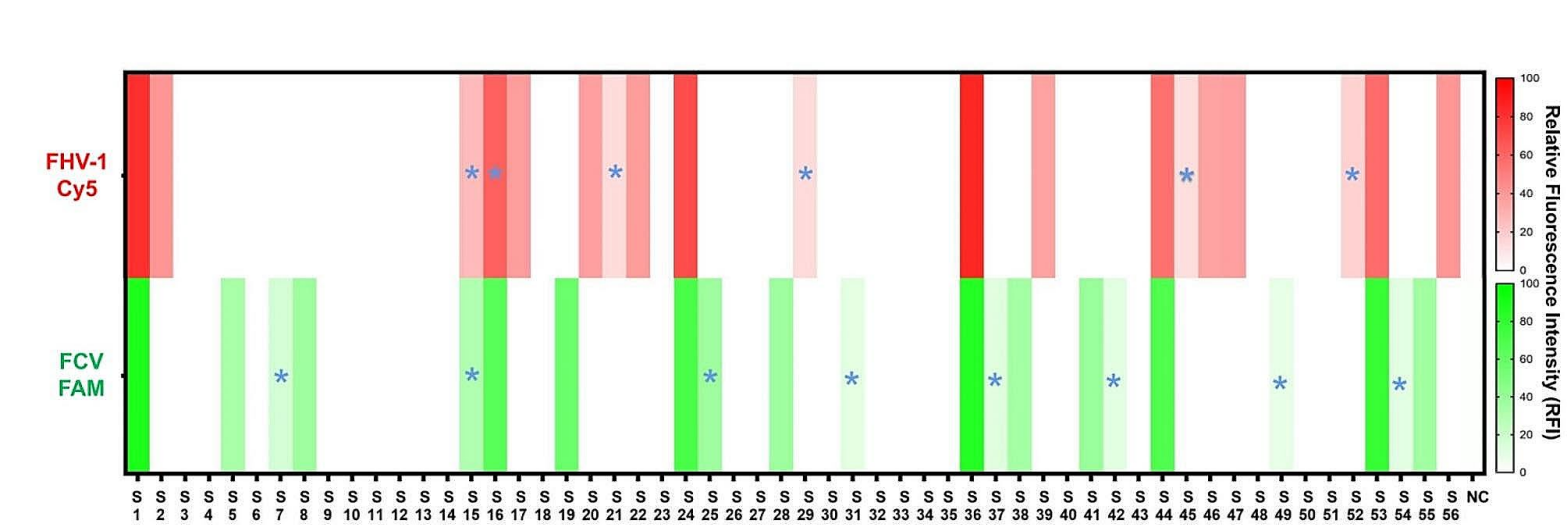
Heat-map of detection results for 56 clinical samples. Current one-tube dRPA-Cas12a/Cas13a-Fluor assay yielded more FHV-1 and FCV positive results compared to the reference real-time PCR methods, when the cut-off Ct value was set as below 36. Additionally, the suspected positive samples yielded by the real-time PCR methods (Ct value was between 36 and 38) were marked by blue stellar. NC: negative control (DNase/RNase-free water)

The clinical utility of one-tube dRPA-Cas12a/Cas13a assay was verified by testing 56 swab samples and comparing the detection results with that of reference real-time PCR methods (FHV-1 qPCR and FCV RT-qPCR). The results of Fluor-based assay and LDF-based assay were in agreement for FCV detection. However, the discordant results of S4, S9, S28, S35 and S44 were observed for FHV-1 detection (Supplementary Figures S5-S7). Based on the Ct values by the reference real-time PCR methods, the Fluor-based assay was considered as a reliable visual modality for FCV and FHV-1 detection. When referring to the default cut-off Ct value (below 36), the positive detection rates of FCV using dRPA-Cas12a/Cas13a-Fluor assay and the reference real-time PCR were 37.5% (95% CI: 24.4–50.6%) and 23.2% (95% CI: 11.8–34.6%), respectively. Similarly, the detection rates for FHV-1 were 33.9% (95% CI: 21.1–46.7%) and 23.2% (95% CI: 11.8–34.6%) using these two methods, respectively, as shown in Fig. 4. Comparatively, the concordance for detection of positive samples (Ct value below 36) between dRPA-Cas12a/Cas13a-Fluor assay and the reference real-time PCR methods was observed. In addition, Sanger sequencing also identified several suspected positive samples (Ct values between 36 and 38) as true positives for FHV-1 (S15, S16, S21, S29, S45, S52) or FCV (S7, S15, S25, S31, S37, S42, S49, S54), as shown in Supplementary Fig. 6. This finding substantially improved the consistency for FHV-1 and FCV detection between these two methods, and generated a portion of FHV-1/FCV positive (12.5%, *n* = 7/56). As a result, the dRPA-Cas12a/Cas13a-Fluor assay demonstrated a sensitivity of 100%, specificity of 100%, and kappa value of 1.00 for FHV-1 and FCV detection (Table l), indicating its exceptional performance in a clinical setting.

## Discussion

In present study, we developed a novel duplex diagnostic method, namely the one-tube dRPA-Cas12a/Cas13a assay, which employed a visual modality for detection of emerging FHV-1 and FCV variants. This approach is easy to interpret and exhibits high sensitivity and specificity, making it a promising alternative for rapid detection in resource-limited settings. Furthermore, by incorporating current streamlined procedures with automated nucleic acid extraction systems, the turnaround time for infield detection can be further reduced to approximate 50 min, thereby meeting the needs of friendly operation for veterinary practitioners. Similar to other molecular methodologies, there will exist a potential risk of off-target detection and subsequent false results due to non-specific amplification and sequence mismatch in clinical specimens [29]. Therefore, meticulous design and selection of oligonucleotides for dual RPA reaction and guide RNA recognition are imperative to ensure accurate diagnosis of the FHV-1and FCV in the one-tube assay.

Notably, the preliminary one-tube assay simultaneously generated detectable signals for both targets without cross-talk, thereby demonstrating the relatively independent detection machinery of Cas12a-crRNA and Cas13a-crRNA complexes in current one-tube reaction system. However, balanced fluorescence signals for both targets were not consistently achieved in unoptimized reaction systems. Previous researchers have endeavored to orchestrate constitutive determinants to enhance the efficiency of multiplex detection, whereas the reaction parameters and components varied in diverse CRISPR/Cas-powered detection systems [23–25, 30]. These findings prompt us to further optimize the reaction settings for current one-tube dRPA-Cas12a/Cas13a assay targeting FHV-1 TK gene and FCV ORF1 gene.

As previously reported, the *trans*-cleavage activity of Cas12a or Cas13a exhibited substrate-dependent activities regarding the amounts of target templates [31]. Therefore, the optimal amount of dual RPA products was determined in present study to maximize detection efficiency for both targets. Additionally, it has been reported that crucial components involved in RNA transcription for the Cas13a-based assay could hinder the capability of the Cas12a-based assay [23, 32], so the optimal concentrations of RNase inhibitor and T7 RNA polymerase mix were also screened for current one-tube reaction systems. Unexpectedly, the remarkable inhibition conferred by RNase inhibitor on Cas12a *trans*-cleavage activity was not consistently observed when its amount exceeded 24 U in the 30 μL of reaction system, possibly indicating a narrow spectrum of concentration-dependent effect for RNase inhibitor, which also requires further study. Based on our previous findings [16, 17], other essential components, such as NTPs, NEBuffer, crRNA, ssDNA, and ssRNA reporters, have been optimized for efficient single RPA/Cas12a-based or RPA/Cas13a-based assay, thus they were kept at fixed amounts in current one-tube dRPA-Cas12a/Cas13a assay. Consequently, high detection performance of hybrid dRPA-Cas12a/Cas13a assay was susccessfully achieved.

Accumulative evidences have revealed that single RPA/Cas12a-based or RPA/Cas13a-based assay can effectively reduce a risk of false-positive detection due to nonspecific isothermal amplification for RPA, thereby achieving high specificity [16, 17, 30, 31]. Accordingly, the current hybrid dRPA-Cas12a/Cas13a assay showed similar advantage in accurate discrimination for specific targets, and achieved high detection sensitivity for both targets in single copy, which is lower than that of multiplex real-time PCR methods [10, 12], NanoPCR method (Nanoparticles assisted PCR) [13], and dual-ERA method (Enzymatic Recombinase Amplification) targeting FHV-1 and FCV [33]. Furthermore, the detection capabilities targeting FHV-1 and FCV were not compromised in the hybrid dRPA-Cas12a/Cas13a assay compared to single RPA/Cas12a-based or RPA/Cas13a-based assay, respectively [16, 17]. In clinical scenarios, several suspected positive samples (Ct value between 36 and 38) were identified by the dRPA-Cas12a/Cas13a assay, and the results were further confirmed by DNA sequencing. However, it should be noted that setting a rigorous cut-off Ct value (below 36) may result in unexpected discordance between dRPA-Cas12a/Cas13a assay and reference real-time PCR methods. In this case, a well-established verification technique should be introduced to discriminate true or false positives when developing a novel methodology. Moreover, discordant results for several clinical samples yielded by LDF-based assay were observed in comparison with Fluor-based assay, which might be attributed to the interference by complex compounds in samples or the possible “hook effect” of colloidal gold-based colormetric modality [34], suggesting the prudent use and interpreation of LDF-based readouts for clinical detecion. In spite of this, exceptional agreement for comparative study indicates high detection performance of dRPA-Cas12a/Cas13a assay, which is reliable and adaptable for accurate diagnosis of FHV-1 and FCV infection in clinical setting.

Although several closed-tube reaction systems have been developed as contamination-free alternatives for single Cas12a-based or Cas13a-based assay [35, 36], which mechanically separate RPA reaction system and CRISPR-Cas reaction system by built-in tube or tube lid, there still exists a concern about the compromised detection capability due to possible thermal instability of constitutive components for Cas12a-based orCas13a-based reaction system that are pre-incubated at 37~39 ℃ for another 20 min in two-step procedure. In addition, a one-step procedure was also applied as another alternative approach, whereas reduced detection capability for Cas12a-based or Cas13a-based assay was observed [36, 37]. This phenomenon may be attributed to the rapid consumption of target templates provided by RPA reaction at initial stage, which will conversely reduce the activities of CRISPR/Cas nucleases [37]. Therefore, it would be wise to deploy a strategy based on rate-limiting crRNA recognition and delayed *trans*-cleavage activation of CRISPR/Cas nucleases [38]. Overall, these findings suggest thatthe two-step procedure is currently a reliable approach for developing one-tube dRPA-Cas12a/Cas13a assay.

However, there are still some limitations to this study. Firstly, the open-tube procedure may lead to nucleic acids contamination, particularly in small clinical lab.Secondly, further study is required to fulfill the discrimination among *herpesvirus* or *calicivirus* lineages originating from other species. Moreover, to accomplish cost-effective and massive in-field detection, it would be beneficial to employ highly integrative approaches with low-cost workflows and high-throughput platforms, such as nucleic acid extraction-free techniques [39], amplification-free techniques [40], and microfluidic platforms [41], which can further simplify the procedures and improve the efficiency of duplex detection. 

## Conclusion

Altogether, the one-tube dRPA-Cas12a/Cas13a assay enables simultaneously ultrasensitive and visual detection of FHV-1 and FCV nucleic acids with user-friendly modality, which provides unparalleled convenience for FHV-1 and FCV co-infection surveillance and decision-making of URTD management.

## Methods

### Recombinant plasmids construction

Nucleic acids of FHV-1 and FCV are prepared using the Tiangen DNA/RNA Isolation Kit (Tiangen, Beijing, China). RNAs are reversely transcribed into cDNAs with PrimeScript™ RT reagent Kit (Takara, Dalian, China) according to the manufacturer’s instructions. The recombinant plasmid DNAs containing inserted target fragments of the FHV-1 TK gene or FCV ORF1 gene are constructed and amplified using established PCR methods, pMD^TM^19-T Vector (TA) Cloning Kit (Takara, Dalian, China) and *E. coli* DH5α Competent Cells (Takara, Dalian, China) as previouly described [16–18].

### Primers and oligonucleotides preparation

**Fig. 5:**
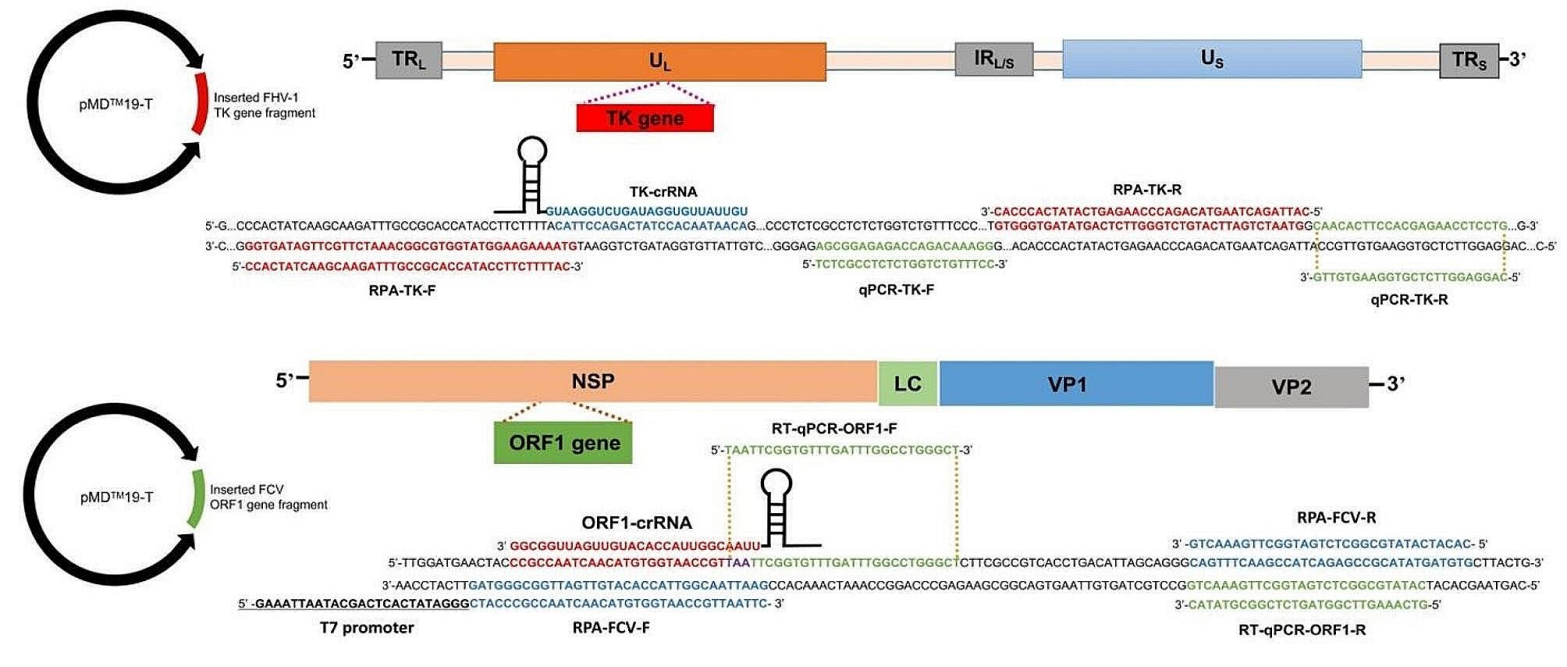
Target sequences recognized by primers and oligonucleotides. The recognition sites of RPA primers, crRNA oligonucleotides, and the reference real-time PCR primers for highly conservative FHV-1 TK gene (66,744 ∼ 66,898 nt, CH-B strain genome) and FCV ORF1 gene (2418 ∼ 2550 nt, SMU-B5-2020 strain genome) are illustrated, respectively. The TK and OFR-1 gene fragments are cloned into pMD^TM^19-T plasmid for later target DNAs preparation

Conservative target fragments of the FHV-1 TK gene and FCV ORF1 gene are screened by genome sequences alignment (GenBank: no. MT813047, MW194990) to design PCR, RPA primers (adding T7 promoter for RNA transcription) and CRISPR RNA (crRNA) oligonucleotides accordingly using Primer Premier 5.0 (PREMIER Biosoft, San Francisco, USA). The crRNA oligonucleotides containing T7 promoter are annealed into dsDNA using an annealing buffer for DNA oligos (Beyotime, Shanghai, China) and HiScribe T7 RNA synthesis kit (NEB, Beijing, China). Then, the synthetic crRNA is digested with DNase I (NEB, Beijing, China) at 37 °C for 15 min and purified with Monarch RNA Purification Kit (NEB, Beijing, China) according to the instruction manual. The concentration of synthetic crRNA is determined using a Qubit 3.0 fluorometer (ThermoFisher Scientific, Waltham, USA). The ssDNA and ssRNA reporters are designed and prepared according to the *trans*-cleavage preference of LbaCas12 (targeting FHV-1 TK gene) and LwaCas13a (targeting FCV ORF1 gene), respectively. All the primers and oligonucleotide sequences, and recognition sites are shown in Fig. 5 and supplementary Tables 1, and synthesized by Sangon Biotech (Shanghai, China).

### Dual RPA reaction

To prepare target nucleic acids, the 50 μL of dual RPA (dRPA) reaction system consists of 1 μL of FHV-1 TK gene template, 1μL of FCV ORF1 gene template, 1.6μL of each RPA-TK-F/R primer (10 μM), 1.6μL of each RPA-ORF1-F/R primer (10 μM), 29.5μL of Primer Free Rehydration buffer (TwistDx, Maidenhead, UK), and 12.8 μL of DNase/RNase-Free Distilled Water (ThermoFisher Scientific, Beijing, China), then 2.5 μL of magnesium acetate (280 mM) is added according to manufacturer’s instructions of TwistAmp® Basic Kit (TwistDx, Maidenhead, UK), and the reaction tube is incubated for 20 min at 39 °C. The RPA products are purified using the UNIQ-10 Spin Column Oligo DNA Purification Kit (Sangon Biotech, Shanghai, China) and determined by gel electrophoresis.

### Hybrid dRPA-Cas12a/Cas13a assay

**Fig. 6:**
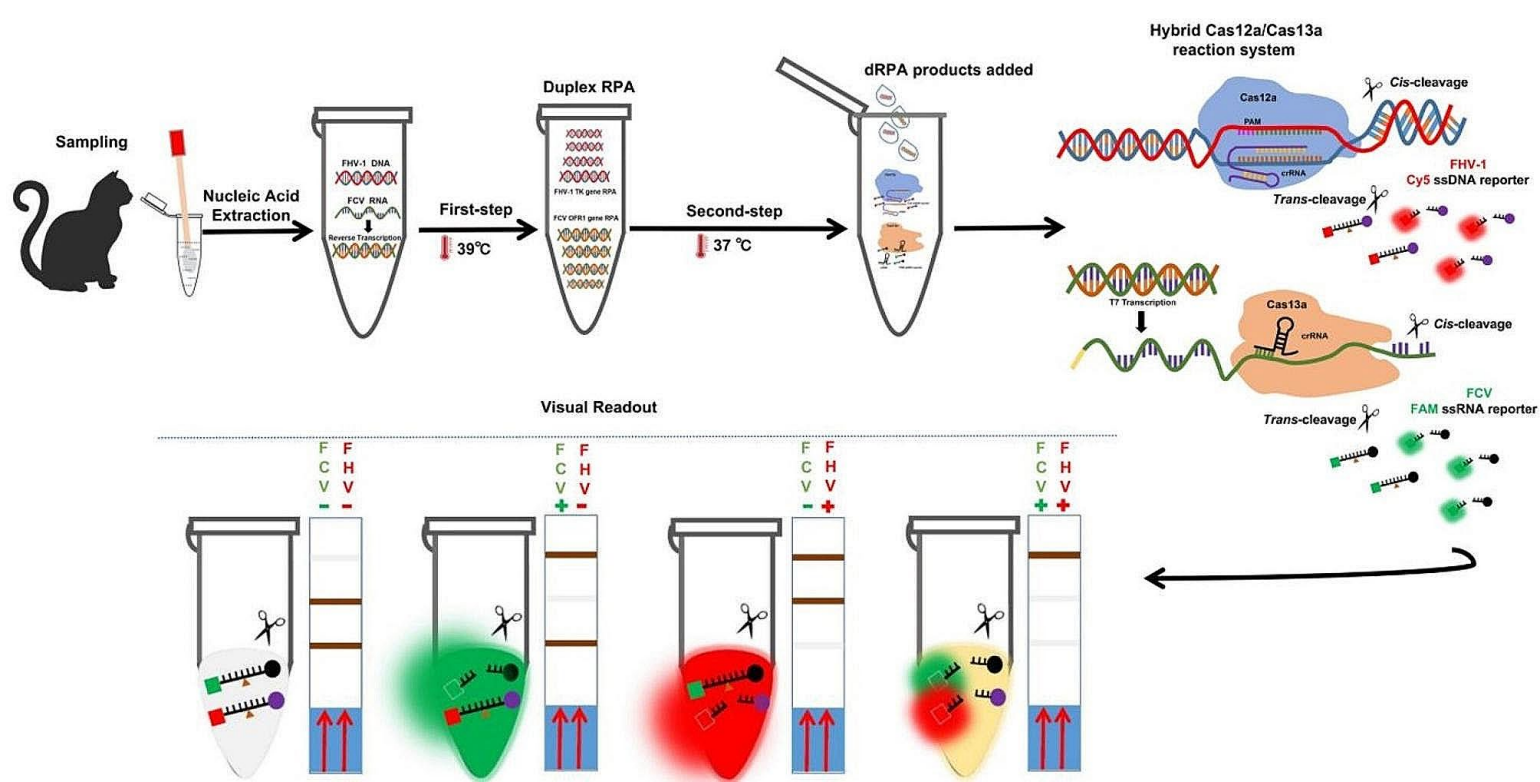
Workflow of two-step one-tube dRPA-Cas12a/Cas13a assay. (**A**) The nucleic acid extracts are prepared from swab samples collected from feline patients with URTD. Reverse transcription is implemented for RNA templates preparation in an independent tube. After that, the target templates are transferred to another tube for immediate duplex RPA reaction (the first step). Then, the prepared dRPA products are added into the premixed hybrid Cas12-crRNA and Cas13a-crRNA complex to trigger the one-tube reaction system (the second step). Before that, the target FCV-ORF1 cDNAs are synchronously transcribed to RNAs for subsequent crRNA recognition using T7 RNA polymerase. In the one-tube hybrid reaction system, Cas12a/crRNA/target DNA oligonucleotides triplets or Cas13a/crRNA/target RNA oligonucleotides triplets are assembled, respectively. Then, hybrid LbaCas12 and (or) LwaCas13a *tran*s-cleavage activities are triggered immediately once specific crRNA-guided recognition of target FHV-1 and (or) FCV templates in the reaction system. Subsequently, the ssDNA and ssRNA reporters are cleaved to generate visible signals. (**B**) Four types of visual readouts based on the dRPA-Cas12a/Cas13a assay are displayed by multi-channel fluorescence detectors or lateral flow dipsticks

The hybrid Cas12a/Cas13a is constructed using two-step strategy, described as one-tube dRPA-Cas12a/Cas13a assay, which combine dual RPA products with hybrid CRISPR-Cas12a/Cas13a *trans*-cleavage activities in a single tube reaction system (Fig. 6 and Supplementary Fig. 8). In the preliminary integrated assay, the reaction system (30 μL in total) consists of 2 μL of dRPA amplicons (initial templates was 10^4^ copies/μL), 30 nM LbaCas12a (NEB, Beijing, China), 3 μL of TK-crRNA (30 nM final), 50 nM LwaCas13a (generated as previously described) [16], 3 μL of ORF1-crRNA(45 nM final), 1.2 μL of RNase inhibitor (40 U/μL) (NEB, Beiing, China), 3 μL of T7 RNA Polymerase mix (50 U/μL) (NEB, Beijing, China), 3 μL of RNAPol Reaction Buffer (10×) (NEB, Beijing, China), 1.25 mM NTP Buffer Mix (NEB, China), 240 nM ssDNA-Cy5/BHQ3 reporter (TTATT) and 240 nM ssRNA-FAM/BHQ1 reporter (poly-14U) and complementary reaction buffer (60 mM NaCl, 40 mM Tris-HCl, 6 mM MgCl_2_, pH 7.3). Then, the integrative reaction system is incubated for 30 min at 37 °C, and endpoint raw fluorescence values are recorded after 30 min in CFX-96 Real-time PCR System (Bio-Rad, Hercules, USA). Subsequently, the reaction tubes are put under the Bio-Rad ChemiDoc MP imaging system (Bio-Rad, Hercules, USA) with its built-in 485 nm (λex) and 643 nm (λex) channels respectively for fluorescence readouts. For the lateral flow dipstick (LFD) readouts, HybriDetect Dipstick 2T (Milenia Biotec, Gießen, Germany) is dipped into the reaction buffer with 20 μL reaction system products containing 200 nM ssDNA-FAM/Biotin reporter (TTATT), 200 nM ssRNA-FAM/Digoxigenine reporter (poly-14U) plus 100 μL hybrid detect assay buffer, and incubated 5 min at room temperature. It is noted that the invisible colorimetric band is determined as positive for the target, and the visible band is determined as negative for the target. The further explanation of LFD readouts is shown in Supplementary Fig. 8.

Considering the possible interference between Cas12a and Cas13a activities in the one-tube system, the crucial components including amounts of dRPA products, RNase inhibitor, and T7 RNA polymerase mix were screened as independent variables, respectively. The visual readouts under the fluorescence detector were quantitatively evaluated to determine the optimal conditions for balanced and maximal fluorescence signals in both channels.

### Specificity and sensitivity testing

For specificity testing, common microorganisms that may cause feline URTD are detected using the one-tube dRPA-Cas12a/Cas13a assay. FCV (SMU-B5-2020, Genbank: no. MW194990), FHV-1 (SMU-2021, sequence not submitted to GenBank), FPV (F-C, Genbank: no. OL547731.1), feline-origin *Escherichia coli* (*E. coli)* (SMU-S120, sequence not submitted to GenBank) and *Klebsiella pneumoniae* (*K. pneumoniae*) (SMU-S-10, sequence not submitted to GenBank) isolates are provided by Key Laboratory of Animal Medicine at Southwest Minzu University of Sichuan Province. Positive samples of *Mycoplasma felis* (*M. felis*), *Chlamydia felis* (*C. felis*), *Bordetella bronchiseptica* (*B. bronchiseptica*), and FCoV are confirmed by a commercial real-time PCR kit (Glinx, Shanghai, China). Then, the DNAs or RNAs of these samples are tested using the one-tube assay.

For sensitivity testing, serial dilutions of plasmid DNAs (10^7^∼10^–2^ copies/μL) with equal amounts of FHV-1 and FCV templates are detected by current dRPA-Cas12a/Cas13a assay and the reference real-time PCR methods [17, 18]. The primers and oligonucleotides sequences used in this study and their matching sites along the virus genome are illustrated in Fig. 5. The dsDNA copy number is determined using the following formula: {[6.02 × 10^23^×dsDNA concentration(ng/μL)×10^− 9^]}/[DNA in length×660].

### Validation of one-tube dRPA-Cas12a/Cas13a assay in clinical samples

Fifty-six conjunctive, nasal and oropharyngeal swabs were collected from cats with URTD, who visited the Veterinary Hospital of Southwest Minzu University, Chengdu City, China, in 2021. The samples are maintained in RNALater™ Viral RNA stable preservation solution (Beyotime, Shanghai, China) for later processing. Then, the nucleic acids of samples are prepared and tested using the dRPA-Cas12a/Cas13a assay (Fluor-based assay and LDF-based assay) and the reference real-time PCR methods, respectively. The real-time PCR is performed on the CFX-96 Real-time PCR System (Bio-Rad, Hercules, USA) using TB Green Premix Ex Taq (Takara, Dalian, China) [16, 18]. The cycle threshold (Ct) values below 36 are determined as the cut-off value for positive results. The Ct values between 36 and 38 are for suspected positive results. The discordant samples between dRPA-Cas12a/Cas13a assays and reference real-time PCR tests are further identified by Sanger sequencing (Sangon Biotech, Shanghai, China).

### Statistical data analysis

Fluorescence readouts and lateral flow dipstick readouts (Supplementary Fig. 8) are photographed by camera and automatically adjusted using ImageJ (NIH, USA). The statistical differences of fluorescence variables are analyzed using the Wilcoxon test or Mann Whitney U test in Prism 8.0 (GraphPad Software Inc, USA). The consistency analysis (sensitivity, specificity, and Kappa value) between the dRPA-Cas12a/Cas13a assay and reference real-time PCR methods is achieved using SPSS 18.0 (IBM, USA).

### Electronic supplementary material

Below is the link to the electronic supplementary material.

Supplementary Material 1

## Data Availability

The data and materials supporting the findings of current study are available in the article and supplementary files.

## Abbreviations

*B. bronchiseptic*: *Bordetella bronchiseptica*
*C. felis*: *Chlamydia felis*
Cas: CRISPR associated proteins
CRISPR: Clustered regularly interspaced palindromic repeats
crRNA: CRISPR RNA
*E. coli*: *Escherichia coli*
FCoV: Feline coronavirus
FCV: Feline calicivirus
FHV-1: Feline herpesvirus type 1
FPV: Feline panleukopenia virus
*K. peumoniae*: *Klebsiella pneumoniae*
*M. felis*: *Mycoplasma felis*
RPA: Recombinase polymerase amplification
RT-qPCR: Real time quantitative polymerase chain reaction
URTD: Upper respiratory tract diseases
