# Supplementary material for: Ultrasensitive and visual detection of Feline herpesvirus type-1 and Feline calicivirus using one-tube dRPA-Cas12a/Cas13a assay
Source: BMC Vet Res. 2024 Mar 16;20:106. doi: 10.1186/s12917-024-03953-9 (PMC10943893; doi:10.1186/s12917-024-03953-9)
Supplement: Supplementary file 1 — Supplementary Material 1 [file 12917_2024_3953_MOESM1_ESM.pdf]

Supplementary Material

Supplementary Table 1 Information of primers and oligonucleotides

Targets	Sequence 5'-3'	Source
PCR-FHV-1 TK gene	Forward: GACGTGGTGAATTATCAGC<br>Reverse: CAACTAGATTTCCACCAGGA	[17]
PCR-FCV ORF1 gene	Forward:TTGGATGAACTACCCGCCA<br>Reverse:CAGTAAGCACATCATATGC	[16]
RPA-FHV-1 TK gene	Forward:CCACTATCAAGCAAGATTTGCCGCACCATACCTTCTTTTAC<br>Reverse:CATTAGACTAAGTACAGACCCAAGAGTCATATCACCCAC	[17]
RPA-FCV ORF1 gene	Forward:T7-*GAAATTAATACGACTCACTATAGGG*CTACCCGCCAATCAACAT GTGGTAACCGTTAATTC<br>Reverse:CACATCATATGCGGCTCTGATGGCTTGAAACTG	This study
qPCR-FHV-1 TK gene	Forward: TCTCGCCTCTCTGGTCTGTTTCC<br>Reverse: CAGGAGGTTC TCGTGGAAGTGTTG	[17]
qPCR-FCVORF1 gene	Forward: TAATTCGGTGTTTGATTTGGCCTGGGCT<br>Reverse: CATATGCGGCTCTGATGGCTTGAAACTG	[18]
TK-crRNA Oligo	TGTTATTGTGGATAGTCTGGAATGATCTACAACAGTAGAAATT*CCCTATAGTG AGTCGTATTAATTTC*-T7	[17]
ORF-1-crRNA Oligo	CCGCCAATCAACATGTGGTAACCGTTAAGTTTTAGTCCCCTTCGTTTTTGGGG TAGTCTAAATC*CCCTATAGTGAGTCGTATTAATTTC*-T7	This study
Fluor-ssDNA reporter	Cy5 /TTATT/BHQ3	This study
Fluor-ssRNA reporter	6-FAM/UUUUUUUUUUUUUU/BHQ1	This study
LDF-ssDNA reporter	6-FAM/TTATT/Biotin	This study
LDF-ssRNA reporter	6-FAM/UUUUUUUUUUUUUU/digoxigenin	This study

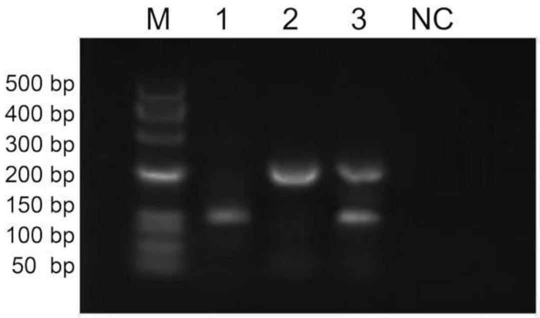

Supplementary Fig. 1 Gel electrophoresis analysis for single RPA and dRPA products. The reaction systems worked effectively for both single RPA and dRPA. M, standard molecular weight; lane 1, ORF1 gene fragment (138 bp); lane 2, TK gene fragment (195 bp); lane 3, dual RPA products (ORF1 and TK gene fragment, respectively); NC, negative control (DNase/RNase-free water).

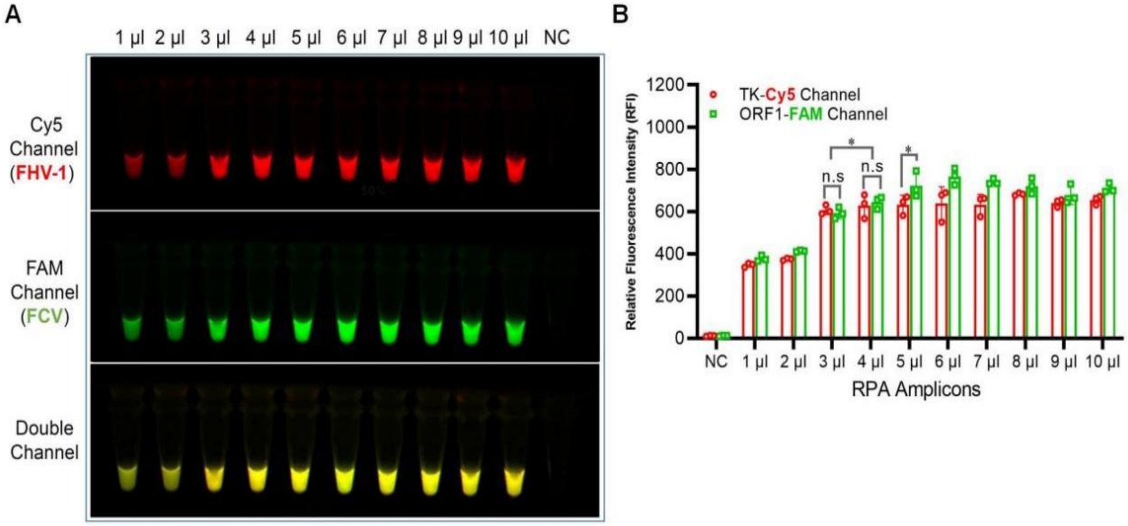

Supplementary Fig. 2 Screening optimal volumetric amount of dRPA products using Fluor-based assay . (A) Accumulating volumes of dRPA products in the preliminary on-tube reaction systems resulted in gradually enhanced fluorescence intensity for both targets. (B) Balanced fluorescence signals were always observed in the preliminary reaction system, and 1 μL or 2 μL dPRA products could not trigger the subsequent Cas12a/Cas13a *trans*-cleavage activities sufficiently until 4 μL dRPA products were added, which was helpful to achieve balanced and sub-saturated fluorescence intensity. Although 5 μL dRPA products generated enhanced fluorescence intensity, unbalanced detection for both targets emerged. NC, negative control (DNase/RNase-free water). Values statistically significant are indicated by asterisks, *P < 0.05; n.s, no significance in statistic analysis.

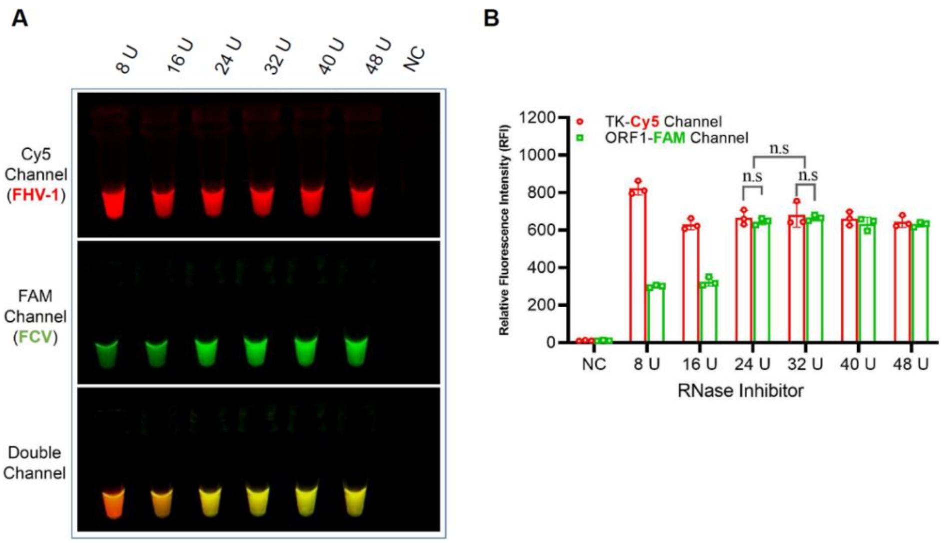

Supplementary Fig. 3 Screening optimal amount of RNase inhibitor using Fluor-based assay. (A) An increasing amount of RNase inhibitor enhanced the fluorescence intensity generated by Cas13a activation (FAM channel), while moderate inhibition of Cas12a activity (Cy5 channel) was observed. (B) Unbalanced fluorescence signals for two targets existed when 8 U or 16 U of RNase inhibitor was added. Then, balanced fluorescence signals for two targets were constantly observed when the amount of RNase inhibitor was up to 24 U in the one-tube reaction system. NC, negative control (DNase/RNase-free water); n.s, no significance in statistic analysis.

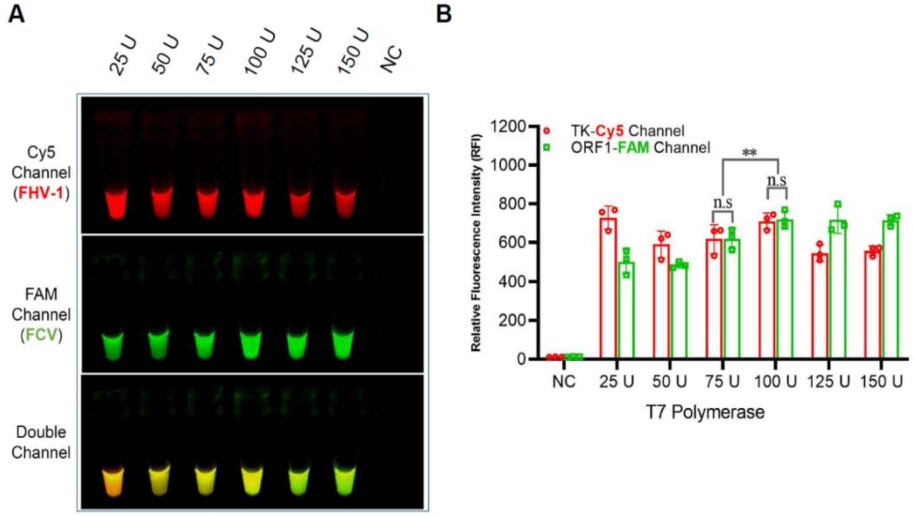

Supplementary Fig. 4 Screening optimal amount of T7 RNA polymerase mix using Fluor-based assay. (A) An increase of T7 RNA polymerase mix enhanced Cas13a activity (FAM channel) and compromised Cas12a activity (Cy5 channel) in a concentration-dependent manner. (B) By contrast, 100 U of the T7 RNA polymerase mix generated higher balanced fluorescence signals than that of 75 U, however, further increase the amount of the T7 RNA polymerase could inhibit the cas12a activity. Values statistically significant are indicated by asterisks, **P < 0.01, n.s, no significance.

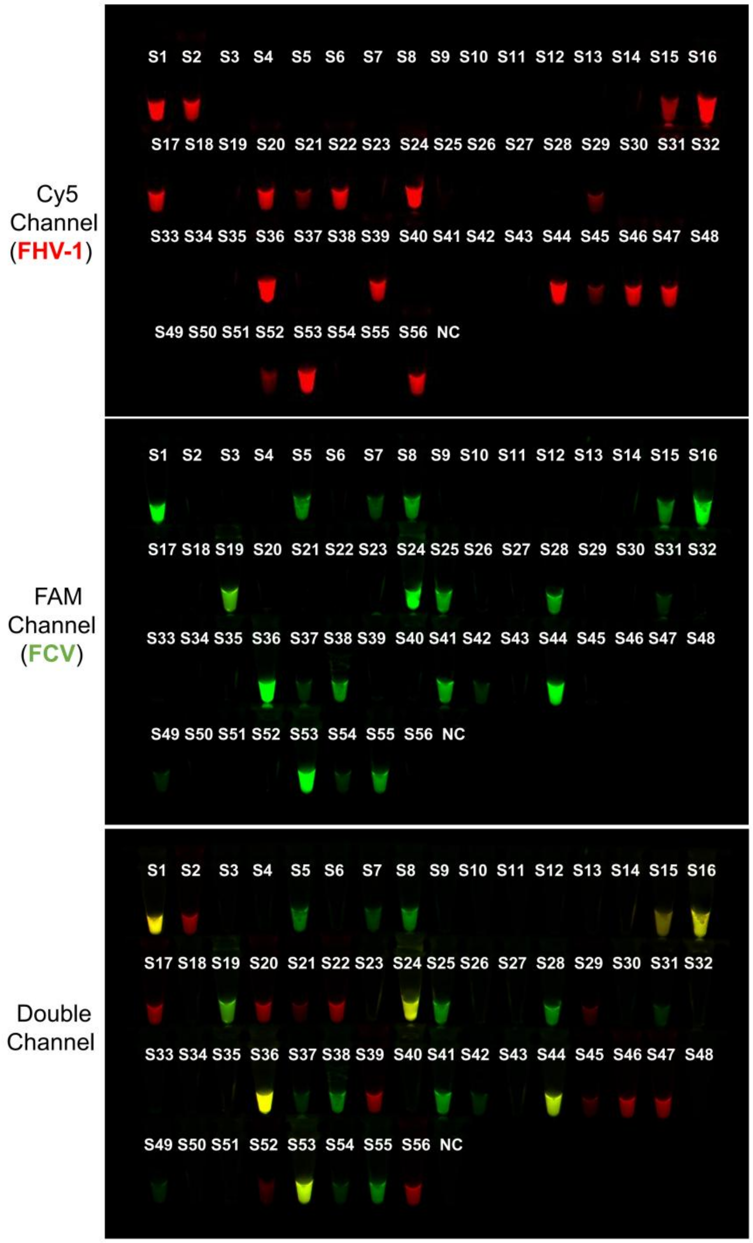

Supplementary Fig. 5 Clinical validation using Fluor-based assay. Visual readouts of FHV-1 TK gene (Cy5 channel) and FCV ORF-1 gene (FAM channel) for 56 clinical samples using a fluorescence detector (Fluor-based assay). Number 1-56, Clinical samples; NC, negative sample.

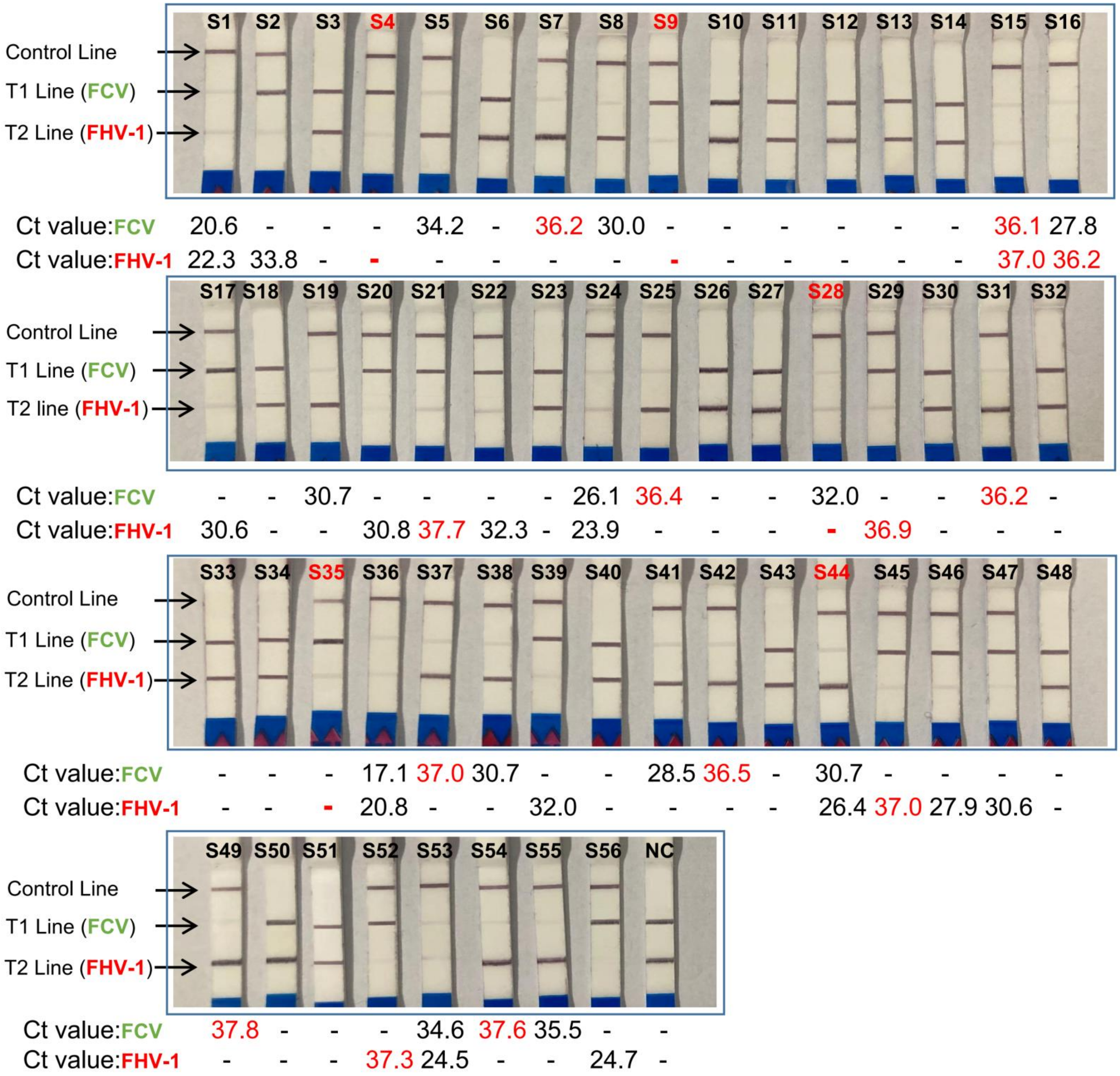

Supplementary Fig. 6 Clinical validation using LDF-based assay and real-time PCR methods. Visual readouts of FHV-1 TK gene and FCV ORF-1 gene for 56 clinical samples using lateral flow dipsticks (LDF-based assay). The corresponding mean Ct values were demonstrated under each dipstick. The Ct values marked in red meant inconclusive and negative samples for reference real-time PCR methods. Number 1-56, clinical samples; NC, negative sample. The visible colorimetric band was counted as positive for the target, and the invisible band was counted as negative for the target. Totally, the detection results of S4, S9, S28, S35 and S44 (marked in red) were discordant with real-time PCR methods.

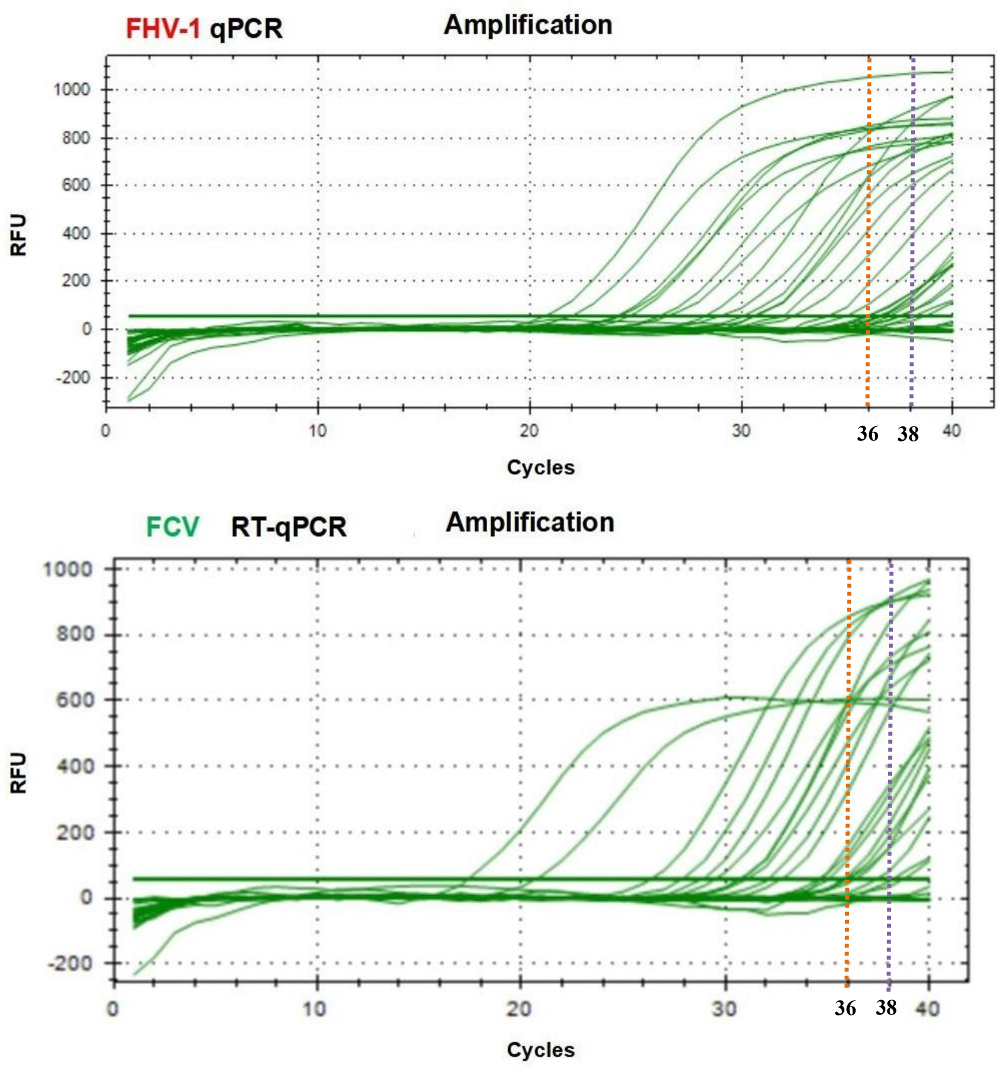

Supplementary Fig. 7 The amplification curves of 56 clinical samples using real-time PCR methods. The targeting FHV-1 TK gene and FCV ORF1 gene were amplified by reference real-time PCR methods, respectively. The rigorous cut-off Ct value for positive was 36 (orange dotted lines) , and high Ct value (36~38) was determined as inconclusive(purple dotted lines).

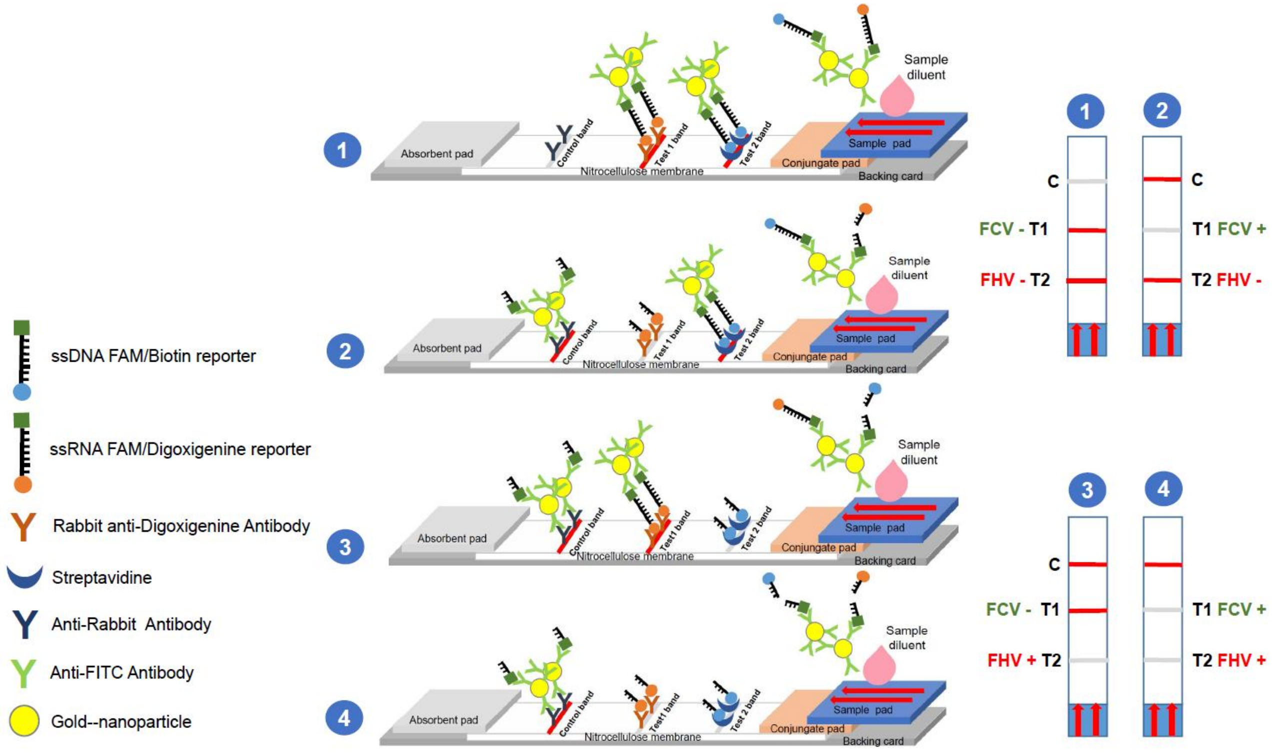

Supplementary Fig. 8 Schematic explanation of FCV and FHV-1 detection readouts on lateral flow dipsticks (LDF-based assay). The CRISPR-Cas12a/Cas13a complexes can detect FCV and FHV-1 molecules on lateral flow dipsticks. When crRNAs (specific for FCV and FHV sequences) recognize the target FCV or FHV amplicons, respectively, the activated collateral nuclease activity of the Cas12a or Cas13a protein results in the subsequent cut off the ssDNA FAM/Biotin reporter (for Cas12a and target FHV) or ssRNA FAM/digoxigenine reporter (for Cas13a and target FCV) in the sample mixtures. The colorimetric signals (produced by the conjunction of gold-nanoparticles on marker bands) due to the separation of terminal labels (FAM, Digoxigenine, Biotin) are detectable with the Milenia HybriDetect 2T® lateral flow dipsticks (Milenia Biotec, Germany). On the lateral flow dipstick, the control band (C) is labeled with anti-rabbit antibody (can combine with antibody conjugated gold-nanoparticles), the T1 band is labeled with anti-digoxigenine antibody, and the T2 band is labeled with biotin ligand (Streptavidine). The synthetic ssRNA or ssDNA reporters preferentially combine with the gold nanoparticles by recognizing the specific antibody labels. If the FCV and FHV amplicons coexist or none of them exists in the sample diluents, the Cas13a and Cas12a will or will not exert their function (separately cut off the ssRNA FAM/digoxigenine and ssDNA FAM/Biotin reporters) leading to the deposition of gold-nanoparticles with fragmentary or intact reporters merely on the control band or two test bands (See Fig. R1 ①④). When only the FCV or FHV amplicons exist in the sample diluents, the Cas13a or Cas12a will cut off the ssRNA FAM/digoxigenine or ssDNA FAM/Biotin reporters, respectively, leading to subsequent retention of gold-nanoparticles with fragmentary or intact reporters on the control band and another test band (See Fig.R1 ②③). Hence, the specific patterns of colorimetric signals on the control or test bands indicate the positive or negative visual readouts for FCV and FHV-1 molecular detection. It is noted that the invisible colorimetric band was counted as positive for the target, and the visible band was counted as negative for the target.
